# NSF DARE—Transforming modeling in neurorehabilitation: Four threads for catalyzing progress

**DOI:** 10.1186/s12984-024-01324-x

**Published:** 2024-04-03

**Authors:** Francisco J. Valero-Cuevas, James Finley, Amy Orsborn, Natalie Fung, Jennifer L. Hicks, He (Helen) Huang, David Reinkensmeyer, Nicolas Schweighofer, Douglas Weber, Katherine M. Steele

**Affiliations:** 1 Alfred E. Mann Department of Biomedical Engineering, University of Southern California, 1042 Downey Way, Los Angeles, 90089 CA USA; 2 Division of Biokinesiology and Physical Therapy, University of Southern California, 1540 Alcazar St 155, Los Angeles, 90033 CA USA; 3 Thomas Lord Department of Computer Science, University of Southern California, 941 Bloom Walk, Los Angeles, 90089 CA USA; 4 Department of Electrical and Computer Engineering, University of Washington, 185 W Stevens Way NE, Box 352500, Seattle, 98195 WA USA; 5 Department of Bioengineering, University of Washington, 3720 15th Ave NE, Box 355061, Seattle, 98195 WA USA; 6 Washington National Primate Research Center, University of Washington, 3018 Western Ave, Seattle, 98121 WA USA; 7 Department of Bioengineering, Stanford University, 443 Via Ortega, Stanford, 94305 CA USA; 8 Joint Department of Biomedical Engineering, North Carolina State University, 1840 Entrepreneur Dr Suite 4130, Raleigh, 27606 NC USA; 9 Joint Department of Biomedical Engineering, University of North Carolina at Chapel Hill, 333 S Columbia St, Chapel Hill, 27514 NC USA; 10 Department of Mechanical and Aerospace Engineering, UCI Samueli School of Engineering, 3225 Engineering Gateway, Irvine, 92697 CA USA; 11 Department of Mechanical Engineering and the Neuroscience Institute, Carnegie Mellon University, 5000 Forbes Avenue, B12 Scaife Hall, Pittsburgh, 15213 PA USA; 12 Department of Mechanical Engineering, University of Washington, 3900 E Stevens Way NE, Box 352600, Seattle, 98195 WA USA

**Keywords:** Conference, Computational neuroscience, Rehabilitation, Adaptation, Plasticity, Personalization, Human-device interactions, Wearables, Big-data

## Abstract

We present an overview of the Conference on Transformative Opportunities for Modeling in Neurorehabilitation held in March 2023. It was supported by the Disability and Rehabilitation Engineering (DARE) program from the National Science Foundation’s Engineering Biology and Health Cluster. The conference brought together experts and trainees from around the world to discuss critical questions, challenges, and opportunities at the intersection of computational modeling and neurorehabilitation to understand, optimize, and improve clinical translation of neurorehabilitation. We organized the conference around four key, relevant, and promising Focus Areas for modeling: Adaptation & Plasticity, Personalization, Human-Device Interactions, and Modeling ‘In-the-Wild’. We identified four common threads across the Focus Areas that, if addressed, can catalyze progress in the short, medium, and long terms. These were: (i) the need to capture and curate appropriate and useful data necessary to develop, validate, and deploy useful computational models (ii) the need to create multi-scale models that span the personalization spectrum from individuals to populations, and from cellular to behavioral levels (iii) the need for algorithms that extract as much information from available data, while requiring as little data as possible from each client (iv) the insistence on leveraging readily available sensors and data systems to push model-driven treatments from the lab, and into the clinic, home, workplace, and community. The conference archive can be found at (dare2023.usc.edu). These topics are also extended by three perspective papers prepared by trainees and junior faculty, clinician researchers, and federal funding agency representatives who attended the conference.

## Introduction

**Fig. 1:**
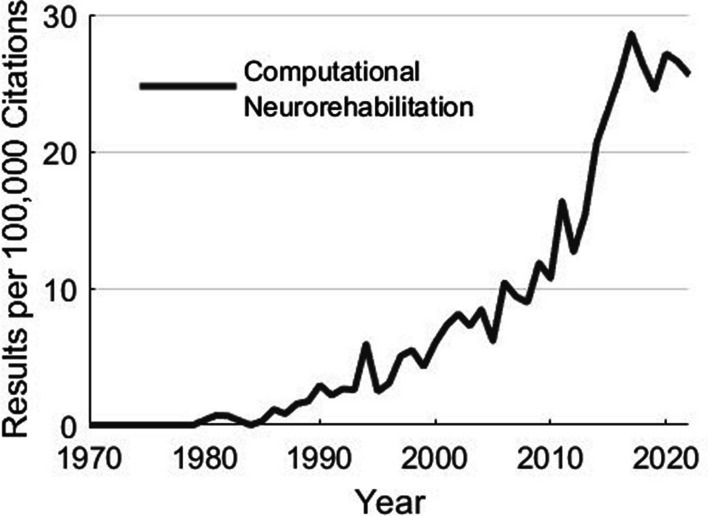
The field of computational neurorehabilitation has grown tremendously over the preceding decades. Usage of the terms “computational” and “neurorehabilitation” in articles indexed by Pubmed. Data generated by Pubmed by Year (https://esperr.github.io/pubmed-by-year/)

Why do we create computational models? Simply put, to help us move from costly and inefficient trial-and-error empiricism towards mechanistic, hypothesis-driven, and evidence-based systematic processes to develop clinical treatments and products [1, 2]. Along these lines, computational modeling has had profound impacts on our scientific understanding. For example, computational models of sensorimotor control and plasticity underlie the design and application of neuromodulatory approaches to enhance motor function during development, aging, and following neurological injury or disease [3–11]. Additionally, computational musculoskeletal models have been used to inform treatment decisions in orthopedics and sports medicine. Computational models describing the trajectories of development, disability, and recovery have the potential to help prioritize and focus treatment. Moreover, models of musculoskeletal dynamics and neural control are regularly used by researchers to design and implement control strategies of assistive technologies [12–18]. The impacts of computational modeling are only set to increase in the coming decades: the emergence of multimodal remote sensors, machine learning, and multiscale datasets (from genomics to behavior) will enable a future in which personalized neurorehabilitation that adapts throughout the course of treatment becomes the norm. In fact, the interest in these areas continues to accelerate (Fig. 1).

As interest and potential impact in these areas accelerates, we convened the *NSF DARE Conference: Transformative Opportunities for Modeling in Neurorehabilitation* that brought together experts and trainees to discuss critical questions, challenges, and opportunities at the intersection of computational modeling and neurorehabilitation. We identified four Focus Areas prior to the conference through discussions amongst the PI team, Advisory Board, and federal funding representatives. These areas were identified as areas of high growth and potential impact for computational modeling in rehabilitation research (Table 1). They represent—in our opinion—pressing challenges and timely opportunities within neurorehabilitation where advances in science, computational methods, and implementation can converge for actionable change. These areas also exemplify the potential for innovation and impact when merging multiple modeling methods (e.g., machine learning and physics-based models) with technology (e.g., exoskeletons or wearable sensors) to support scientific understanding, target neurorehabilitation outcomes, and improve quality of life. These areas are highly synergistic with NSF’s *Disability and Rehabilitation Engineering Program* and NIH’s *2021 Research Plan on Rehabilitation*.

In this paper we summarize and comment on the perspective and insights from the expert community assembled to help establish a foundation by which computational modeling can create the scientific directions, theories, and actionable platforms to improve the efficacy and personalization of neurorehabilitation. This is based upon discussions with participants during the meeting in general, and among the co-authors during the writing of this paper. Additional concluding remarks are included in the three other companion papers that reflect key take-away points from other conference constituents (e.g., trainees, federal funding representatives, and clinician scientists). Mirroring the structure of the meeting, we now visit and comment on each focus area. It is important to note that the main conclusions are summaries drawn by the authors. As such, they represent the viewpoints of the authors, and not a consensus process of conference attendees. The companion papers further clarify this distinction where each set of authors provides their point of view and provide further conclusions and key take-aways from other constituent groups who participated in the conference (e.g., trainees, clinician scientists, and federal funding representatives). Those additional three papers in this same issue are titled, respectively:

- NSF DARE—Transforming Modeling in Neurorehabilitation: A Patient-in-the-Loop Framework
- NSF DARE—Transforming Modeling in Neurorehabilitation: Clinical Insights for Personalized Rehabilitation
- and
- NSF DARE—Transforming Modeling in Neurorehabilitation: Perspectives and Opportunities from US Funding Agencies

**Table 1:** Focus areas for the DARE 2023 conference

**Focus area**	**Description**
Modeling adaptation and plasticity	How do we leverage modeling to understand neuroplasticity? How can the integration of novel imaging technologies, machine learning, and physiology-based models be used to understand the complex processes underlying beneficial neuroplasticity and adaptation that support learning and recovery?
Modeling for personalization	A central challenge in rehabilitation is that each individual’s developmental, injury, treatment response, and long-term recovery trajectory is unique. However, it is necessary to first determine the degree of personalization required to optimize and support development and recovery in practice. How can we leverage large, diverse, and real-time datasets to support an appropriate and effective level of personalization to optimize outcomes?
Modeling human–device interactions	Interconnected human-centered technology has become a critical part of function and rehabilitation—including development, acute care, training, and activities of daily living. However, neurorehabilitation requires new engineering approaches to support the design, interaction, integration, and control of devices to support development and recovery. How can modeling inform and accelerate this development?
Modeling ‘in-the-wild**’**	Rehabilitation does not end at the clinic’s door – it extends into and is meant to serve our daily lives and practices. How can modeling support rehabilitation in unstructured environments to offer actionable insights to support and promote recovery? How can modeling identify and help dismantle environmental and societal barriers that cause disability, as well as support and enhance activities of daily living?

## Modeling adaptation and plasticity

### Brief background

Adaptation and plasticity are central to our nervous system’s ability to acquire new abilities, adapt them to changing situations, and recover function after injury. The role of synaptic plasticity in sensorimotor learning and adaptation is the subject of much work described in several reviews [19–22]. Here, our main interest is the role of plasticity in the recovery of function after injury for neurorehabilitation. For example, after stroke, neurons re-wire connections both immediately surrounding the injury and across distant brain areas [23–28], and these changes in connectivity are correlated with improvements in function with and without rehabilitation [27, 28]. Critically, adaptation and plasticity are not guaranteed to be fast, well-guided, or beneficial (i.e., adaptive plasticity). Plasticity can also be maladaptive [29, 30], a neurorehabilitation analog to focal dystonias [31, 32].

A key goal of neurorehabilitation therapies is to promote plasticity mechanisms that improve function while also mitigating maladaptive changes to neural circuits. Many therapeutic approaches have been proposed and attempted to achieve this goal—with varying degrees of success—ranging from targeted behavioral training to implantable devices that stimulate neural circuits.

Computational modeling of adaptation and plasticity can provide paths to maximize the impact of neurorehabilitation therapies [3, 33]. The plasticity and adaptation that occurs after injury span many levels of the nervous system, from cellular processes (e.g., changes in ion channel expression) [34], to network interactions (e.g., changes in synaptic connections) [27], to behavioral changes (e.g., compensatory strategies) [35]. As a result, a wide range of modeling methods have been used to describe plasticity at different levels [36]. In lieu of an exhaustive survey of existing models, we highlight some useful categories of model types. For a particular phenomenon, such as synaptic plasticity, models may focus on different levels of abstraction. For example, phenomenological models like Hebb’s rule describe input-output relationships between the rate/timing of neural activity and connection changes, describing the computational principle without directly modeling the biological implementation [37]. Biophysical models of spike-timing dependent plasticity, in contrast, describe the physiological changes within neurons that give rise to synaptic changes [38]. Data-driven models (i.e., machine learning) have also been employed for modeling a variety of plasticity phenomena (e.g., [39]).

A variety of model types, spanning different levels of the nervous system, have been used to describe how the nervous system will respond to an intervention to inform rehabilitation therapies [3, 40]. Mechanistic models of how behavior evolves as we adapt to altered dynamics like a split-belt treadmill, for example, informed training interventions to improve gait post-stroke [41]. Phenomenological Hebbian plasticity models informed stimulation protocols to increase the functional connections among regions in the brain [11, 42, 43], and from the brain to muscles or spinal circuits [44–47]. Models describing nervous system changes over time are also valuable for predicting outcomes and to guide clinical decision-making. Many examples of these models rely on data-driven discovery from large datasets. For example, the increasing prevalence of neural imaging technologies in clinical practice have led to large datasets to develop algorithms that predict functional recovery after stroke [48]. Machine learning approaches have also been used to assess whether devices like non-invasive brain-computer interfaces will be effective [49, 50] and optimal parameters for therapies like deep brain stimulation [51].

These examples highlight the diversity of plasticity models and applications in neurorehabilitation. As with any other computational modeling effort [2], decisions must be made about the level of abstraction and detail. The challenge of these decisions is acutely clear in the realm of adaptation and plasticity, where mechanisms span spatial scales from synapses to behavior, and timescales from milliseconds to months [20]. Many existing models used for neurorehabilitation focus on a single scale (e.g., describing behavioral changes). Models that bridge neurological mechanisms of plasticity to behavior will likely be needed to improve the precision of neurorehabilitation therapies. Such models will require cross-disciplinary collaboration to develop and validate. Similarly, most existing models focus on describing a particular time-point, such as functional recovery after a certain time with a particular, static therapy. The many time-scales of adaptation and plasticity present challenges for modeling overall trajectories, including the impact of interventions and changes in treatment over time [52].

Extending models of plasticity to span spatial and temporal scales could open new ways to harness the power of computational methods in neurorehabilitation. The dynamic nature of the nervous system creates a variety of challenges for building therapies. Assuring an assistive device provides meaningful functionality for extended periods of time requires characterizing plasticity that may occur in response to the device and developing devices that can adapt accordingly [53]. Similar considerations are needed for therapies where protocols may need to adapt over time as abilities change [54]—a form of meta-adaptation that mirrors meta-plasticity (i.e., ‘plasticity of plasticity’ [36]). Achieving the goal of smart, personalized, and adaptive neurorehabilitation therapies will require models that can capture the dynamics of plasticity processes as well as human-device interactions that will influence those dynamics. This will require new approaches to bridge across models that predict how the nervous system will change in response to a given intervention and those to describe how interventions inpact the trajectory of changes in the nervous system and changes in behavior over time.

### Commentary

The DARE workshop highlighted many fundamental challenges and opportunities in modeling adaptation and plasticity for rehabilitation applications. Multiple presentations (see Appendix for speaker summaries) speak to the promise of using computational models to disentangle diverse learning mechanisms used by the nervous system (e.g., Roth, Mariscal). Mechanistic models such as those used by Roth shed light on the neurophysiological underpinnings of disorders. Their findings, for instance, suggest that Parkinson’s disease can lead to deficits in a single learning mechanism while leaving others intact. Similarly, data-driven methods to identify components of learning used by Mariscal allowed them to characterize how learning generalized to new contexts more precisely than past studies. A critical next step missing from most workshop submissions is using these model-derived insights to directly guide clinical therapies. Future work towards these efforts will have to contend with challenges closely related to those faced in personalization efforts (see below). For example, do models and their parameters need to be estimated on populations of people or individuals? Models may also require updating over time as learning proceeds, closely mirroring challenges faced in human-device interactions.

Multiple presentations (e.g., Liew, Orsborn, Hight, Schwock) aimed to characterize plasticity that occurs as the result of therapies and interventions or injuries. Schwock’s work highlights the potential benefits of computational models to quantify changes between regions of the nervous system when they are embedded within a large network (see also [55]). This work highlights the challenge of identifying the most useful measures of nervous system plasticity, since nearly all metrics will be approximations. Data-driven studies probing how physiological measurements relate to clinical outcomes will likely be critical to identify the most useful experimental and computational measures of plasticity for neurorehabilitation. Collaborations between researchers developing novel assays of plasticity and those using large clinical datasets to predict clinical outcomes, such as discussed by Liew, will be invaluable for future research and translation. Though such collaborations will likely involve navigating the challenges of measurement feasibility, such challenges highlight the potential promise of extending neurorehabilitation ‘in-the-wild’ (see below) and research into quantifying plasticity metrics.

Designing interventions that induce plasticity is central to any rehabilitation effort. Data-driven predictive models, such as those developed by Liew, provide methods for predicting how someone may respond to an intervention dose. However, these models have largely been used to predict a single endpoint, which may miss dynamic interactions between plasticity and an intervention, as highlighted by other presentations (e.g., Orsborn, Hight). Research with brain-computer interfaces and cochlear implants demonstrate that even interventions that intend to replace a function (rather than rehabilitate) induce plasticity. This plasticity may be influenced by how the device is designed (e.g., Orsborn’s investigations into co-adaptation with brain computer interfaces), and could be further manipulated by purposeful device interventions (e.g., vagus nerve stimulation presented by Hight). User-device interactions to shape plasticity open a huge opportunity to shape plasticity for rehabilitation. Capitalizing on this opportunity, however, will require improving models of how devices induce plasticity. Translating methods to shape plasticity with devices into meaningful clinical therapies will also require methods to predict functional outcomes.

Beyond these examples of scientific challenges, we also noticed important challenges and opportunities to create the scientific community needed to tackle these challenges. All talks focused on plasticity, but we were particularly struck by the topic diversity. For instance, the presentations spanned upper limb movements (Roth, Orsborn), locomotion (Mariscal), clinical sensorimotor function assessments (Liew), and hearing/speech (Hight). There was also a diverse range of methods used to quantify plasticity, from behavior (Roth, Mariscal, Liew), clinical neuroimaging (Liew), and high-resolution electrophysiology (Orsborn, Schwock). This breadth fostered rich discussions across sub-fields that do not regularly interact. Integrating the knowledge gained from this diversity of methods and applications and refining models of plasticity and adaptation for rehabilitation will require bridges across these communities and translating terminology between fields.

## Modeling for personalization

### Brief background

**Fig. 2:**
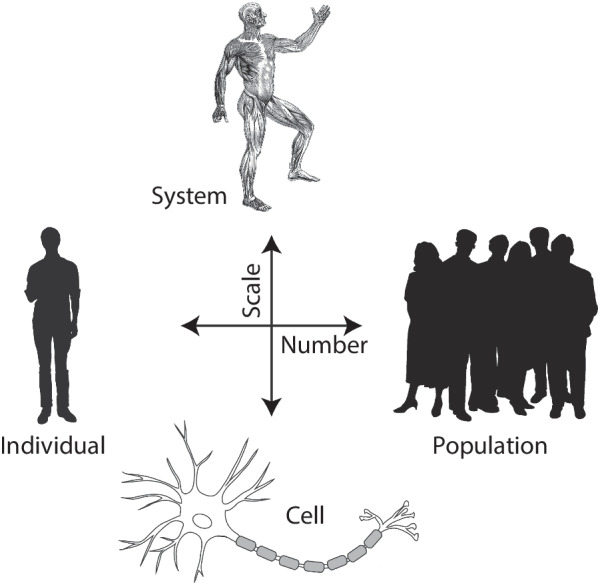
The personalization spectrum for a particular population across physiological scales and numbers of individuals. Models at a given scale that are assumed to characterize a specific individual are often called ’patient-specific’, and population models ‘generic’ as they are assumed to apply to many individuals. Free images adapted from Clipart Panda, Muscular Systems, and pngegg

Computational models of neuromuscular function for neurorehabilitation can be used to aid the clinical (i) classification, (ii) explanation, and/or (iii) prediction at any or all of the stages of patient intake, treatment, or follow-up. While there are multiple computational modeling approaches and techniques [2], they often fall into the two broad categories of statistical or descriptive vs. mechanistic models, both of which are, in George E.P. Box’s words, ‘useful fictions’ [56]. What is personalization in this context? Importantly, the degree of personalization is in fact a spectrum of granularity: from a particular ion channel, cell or neuron, to a neural circuit, to an individual, to a subset of individuals, to a particular population (Fig. 2). As per Occam’s Razor, modelers should aim to model at the coarsest necessary level with the fewest number of assumptions to ask and answer questions about function, recovery, or interventions in a useful and mechanistic manner.

There have been long standing debates on whether both statistical and mechanistic models could, or should, be generic (i.e., apply to the entire population) vs. patient-specific (i.e., apply to a single individual). From this perspective, the current discussion about personalized models, ‘digital twins’, personalized medicine, etc. is simply the latest iteration of this long standing debate that can be traced back to the epistemological origins of clinical diagnosis (which assumes multiple patients can be thought of as having the same disease), clinical trials (where all patients are expected to have an average response to a same treatment), and biomechanical modeling (where a model can in principle represent a given population or individual well-enough). What is often not stated explicitly about digital twins is the degree to which they are aspirational, because they are, by construction, difficult to develop and impossible to validate. They inhabit the bottom left corner of the personalization spectrum as they should apply to a specific individual and be accurate to a sufficiently small scale to capture the physiological processes relevant to the clinical question. To bring clarity to these longstanding questions and their recent iterations, without claiming to resolve them, it is important that we be clear on what a computational model is, and how this informs our efforts to achieve modeling for personalization.

A computational model for neurorehabilitation is, at its best, a mathematical or numerical representation of hypotheses about function, dysfunction, and/or response to treatment. Thus, it is important to explicitly distinguish between a model’s topology vs. its parameter values [2, 40, 57]. The topology of a computational model is its structure (at the appropriate scale) explicitly defined by the type, number, and organization of the elementary ‘building blocks’ and their interactions. The parameters values are then the particulars associated with each building block and their interactions. The hypothesis can be cast in the form of patterns or associations (for statistical models) or principles at work (for mechanistic models).

In the case of the broad category of statistical models, a data set is used to tune the parameters of a given mathematical representation of relationships (e.g., linear regression, principal components analysis (PCA) , artificial neural networks (ANNs) via, respectively, slope and intercept, PC loadings and variance explained, and weights among nodes and layers). Statistical models are often called ‘black-box’ models, because the model topology and its parameters have little or no direct physically or physiologically evident relationship between cause and effect. That is, they can be considered to be unencumbered by a hypothesis—if by hypothesis we mean a mechanistic explanation of a phenomenon. While the term ‘prediction’ is often used in statistical models (e.g., a strong and significant linear regression allows feature *x* to ‘predict’ feature *y* in a given population), this does not imply causality. Nevertheless, statistical models are useful, have their place, and are popular because they can be quite powerful in finding unplanned associations by using all features in large data sets.

The topology of mechanistic models, in contrast, is the unambiguous statement of the assumed physiological mechanisms and the causal relationships among them (i.e., a formal hypothesis). For example, computational models can be based upon modules simulating muscles, the spinal cord, and/or the control of movement. However, modelers very quickly find themselves having to make difficult decisions about modeling scope and level of detail—which are determined by the experience and skill of the modeler, the computational resources available, and the clinical question being asked. These decisions also directly define and affect the ability of the mechanistic model to be personalized. These modeling choices have naturally led to valuable debates about choosing ‘simple’ vs. ‘complex’ models. The dilemma across levels of complexity is that more detailed models are more personalizable (i.e., they have a greater number of free parameters to adjust to match a given individual), but require more and better knowledge of mechanisms—and experimental data to validate their more detailed topologies and greater number of parameters.

### Commentary

Considering that ‘personalization’ is a spectrum across scales and numbers of individuals (Fig. 2) what we need are models within that spectrum that provide good-enough assessments of impairment for a given patient (or population) to improve recovery [58–60]. From the literature and outstanding presentations at the conference (see Appendix), we were reminded that for successful personalization, one must be clear about the goal or targeted level of function and follow through with appropriate statistical or physics-based models with topology and parameters that suffice for a specific clinical need. For example, human-in-the-loop-optimization is a field that is becoming practical, as demonstrated by Collins’ work on adaptable exoskeletons. This allows guiding the interactions among the optimization towards improving the client’s function by controlling the hardware-algorithm-human dynamical system (which is often difficult to build). Saul made a clear case for the level of personalization needed to achieve useful results. Patton reminded us that personalized assistive devices and approaches need not be complex actuated devices. Computational modeling, which can be complex, can be used to create personalized devices that use simple, passive elastic elements. In fact, such devices are able to reach much greater numbers of users world-wide than powered, computer-controlled versions. Lajoie underscored how the within- and inter-subject variability of response in neurostimulation (both in mapping stimulation location and parameters at a given location) continues to be a critical bottleneck that prevents effective personalization of neurorehabilitation. However, they demonstrated a framework requiring few data points (i.e., few-shot) to adapt the response to neurostimulation that would enable effective personalized neurostimulation (a form of meta-learning, or learning to learn concepts quickly [36]). Allen presented evidence that computational methods to extract motor coordination strategies fare better at capturing the control of walking when they also include the control of whole-body balance. Finally, Lin emphasized how critical it is to collect outcome measures at the point of care that are relevant to treatment. Moreover, carefully chosen outcome measures available in the clinical setting can have meaningful and neurologic underpinnings that could enhance the utility of computational modeling in neurological conditions and stroke.

The amount of data needed to personalize a given therapeutic approach is a common thread in all approaches presented. While the distinction between generic and patient-specific models can be seen as a dilemma in terms of the amount of data needed, one can argue it is, in fact, a false choice. One possible way out of this dilemma can be found by combining mechanistic and statistical approaches to create data-driven clusters of a finite number of models (i.e., stochastic models) [57] that allow a meta-learning few-shot framework. Similarly, available data to fit models in the form of repeated clinical tests have been typically sparse, leading to models that were necessarily simple to avoid overfitting [8, 61] (see [40]). Compounding the problem is that parameter estimates are often based on ‘point estimation’ methods such as least-squares to fit average or individual data. However, recent developments in Bayesian modeling, e.g., [52], seamlessly generate credible intervals for these predictions and can incorporate prior information (e.g., parameter mean and variance) from previous studies to further improve individual predictions. In addition, Hierarchical Bayesian modeling, which involves simultaneous determination of the population parameters, as well as the individual-level parameters given the data from all participants, can improve predictions when little data is available for new patients by ‘borrowing’ knowledge from previous patients—see [52] for a recent example.

As a final comment, we would be remiss if we did not mention the undergoing revolution in machine learning that Transformers and their variants (e.g., Large Language Models (LLMs) and Generative Pre-Trained Transformer (GPT)) that rose to prominence c. 2017 [62] and are now accelerating exponentially. There are exciting novel opportunities for this technology in all Focus Areas (Table 1). As a particular example, it is important to know what this technology has the very promising ability to bridge the gap between generic and patient specific computational models. An instant classic is the example of ChatGPT’s ability to ‘learn’ a language, and then tailor sentences to the style of writing of a given author [63], which is a form of transfer learning from generic to particular. Such approach is already being applied to, for example, training a transformer on numerous examples of brain structure to then detect a patient-specific anomaly (i.e., a tumor) in spite of inter-subject variability [64].

## Modeling human–device interactions

### Brief background

Devices are ubiquitous in our everyday lives. Since the advent of simple tools and machines such as the lever, wheel, and pulley (as well as assistive devices like canes, pouches, and wheelchairs) humankind has developed increasingly complex assistive devices to reduce the physical effort and time required to move and support ourselves and objects in our environment. Assistive devices have a long history since prehistoric times, with Egyptian hieroglyphs showing the use of staffs and canes [65] through medieval innovations like Gottfried von Berlichingen’s development of the iron hand in 1504 [66]. Since the middle of the past century, robotic devices have been formally proposed as potentially transformative therapeutic tools for physical rehabilitation [67–70].^1^ Today, rehabilitation robotic devices can be configured to allow for precise control of the position and orientation of select body segments or the load applied to them, and this can be done over many repetitions without marked changes in the robot’s performance. As a result of these capabilities, rehabilitation roboticists and therapists recognized the opportunity by which devices could potentially offload some of the physical demands that therapists encounter during taxing rehabilitation interventions such as body-weight supported treadmill training [71, 72]. Robots can also facilitate the repetitive, task-specific practice necessary to provide the dose needed to drive motor learning via adaptation and plasticity and, if integrated with game-based interfaces, also provide a means by which therapy can be made more engaging [73]. Outside the clinic, devices such as powered wheelchairs, exoskeletons, and prostheses can act as assistive devices that compensate for weakness and potentially increase overall activity levels [74]. Due to the large number of potential devices and parameters, there is an urgent need to use modeling, among other approaches, to move away from empiricism in the design and operation of such devices [1].

When considering the design and ultimate real-world use cases of devices in rehabilitation, one must determine an appropriate engineering control strategy that aligns with the theoretical and physiological basis for a given therapeutic intervention. Early rehabilitation robots relied on ‘position control’ to guide the user’s limbs through prescribed trajectories [72] based, in part, on animal studies which showed that passive movement could engage spinal circuits involved in behaviors such as walking [75]. While there may still be select applications in which this control strategy is appropriate, it is often inadequate because patients need to be actively involved in rehabilitation, but naturally ‘slack’ if a robotic device is allowed to do all the work of moving the limb for them [76]. Assist-as-needed algorithms partially address this concern by only providing assistance if the client also exerts a given level of effort and does not become a free-rider [77]. A key element that is often overlooked when designing and evaluating devices for rehabilitation is how sensory deficits, which are particularly common but often difficult to assess, impact the efficacy of training. Additionally, we still lack a strong theoretical basis upon which we can personalize the dose of robotic rehabilitation interventions, though Schweighofer and others are actively developing statistical modeling approaches to forecast long-term recovery patterns [52].

Devices used to facilitate rehabilitation are, of course, not only confined to robotics. Both passive and active implantable devices are also critical elements of the rehabilitation continuum and present unique challenges not present with devices that are picked up and used, or worn such as exoskeletons that can be donned and doffed. Implanted joint replacements and osseointegrated prostheses rely on a semi-permanent or permanent physical interface between a synthetic device and the musculoskeletal system [78]. To be successful, surgeons must choose the correct implant for a patient’s unique anatomical structure, and once the device is implanted, they often rely on past experience to estimate the level of function and quality of life after surgery [79]. Traditional imaging methods such as X-ray and MRI can be used to create digital, anatomical models to improve the fit of implantable devices, but these methods are currently unable to determine how the surgery and subsequent recovery will affect the afferent feedback from the joint or the joint’s mechanical properties. Thus, computational models that integrate a finite element representation of the device along with dynamic models of local tissue properties and their changes over the course of recovery could potentially transform the field.

Neuromodulatory devices have become a critical tool for both assessing neuromotor function and delivering neurorehabilitation interventions. These devices can stimulate regions of the central and peripheral nervous system using either direct current or by generating magnetic fields to induce current in underlying neurons. Non-invasive transcutaneous stimulation of peripheral nerves is often used to measure nerve conduction velocity and test for the presence of peripheral neuropathies [80]. Similarly, transcranial magnetic stimulation has been used to estimate the functional integrity of corticospinal tract [81], and these measures of integrity, as measured by the size of stimulation-evoked electromyographic responses, form a central element of the PREP algorithm for predicting motor recovery in people post-stroke [82]. Invasive techniques such as deep brain stimulation have become indispensable elements of neurorehabilitation for people with Parkinson’s disease as these devices help alleviate motor symptoms such as tremors and bradykinesia [83]. More recently, stimulation of the vagus nerve via implanted electrodes has shown promise for improving upper limb function in people post-stroke [84], presumably via mechanisms that include reduced systemic inflammation, heightened angiogenesis, and improvements in axonal regeneration [85]. Together, this broad spectrum of devices that support neurorehabilitation and daily function represent an area of high need and impact for computational modeling.

### Commentary

One of the central themes that emerged during the DARE Conference was the need for both basic scientific studies and computational models to augment sensation for prosthesis users and other assistive technology. Commercial prostheses typically lack a means by which users can accurately perceive tactile information about the physical interaction between the device and the external world. If this information could be provided to the user through appropriate afferent channels, it may be possible to dramatically improve their ability to manipulate objects with an upper limb prosthesis or improve balance control when this information is integrated into lower limb prostheses. However, the speakers at the meeting (see Appendix) noted two key areas of opportunity in this space. First, they acknowledged the need for precise computational models capable of encoding information about the mechanical interaction between the prosthesis and the external world. Second, they highlighted the need for computational models to inform decisions about how and through which afferent channels this information should be transmitted to the user.

The long-term effectiveness of devices for neurorehabilitation relies on the assumption that humans can adapt their sensorimotor control strategies to acquire the potential benefits of a given device-driven intervention. For decades, neuroscientists and engineers have focused on developing mathematical representations of the learning processes (see section on Adaptation and Plasticity) at play during human-device interactions [86–88], and this knowledge has been used to design control strategies for rehabilitation robots [89]. However, while models of sensorimotor learning often focus on aspects of learning thought to be mediated by supraspinal structures and cortico-spinal pathways, many neuromotor impairments result in hyperexcitability (and/or inhibition) of subcortical structures and brainstem and proprio-spinal projections to $\alpha$ and *gamma* motoneuron pools, resulting in incorrect voluntary and/or undesirable involuntary responses when reacting to imposed movement or loads from robotic and wearable devices [90, 91]. Computational models will be indispensable for understanding how distributed networks throughout the central and peripheral nervous system influence performance and learning in health and disease.

Another major challenge that remains for developing computational models of human-device interactions is that one would often like to know what objective(s) drives an individual’s behavior as they interact with a device, but this is an ill-posed, inverse problem, as a given behavior could be ‘best’ for an infinite number of potential objective functions. There remains a need to develop new approaches to estimate the objectives that drive our behavior beyond work that focuses on minimizing energy cost or performance errors so that rehabilitation interventions can be better aligned with the patient’s explicit (e.g., walking faster) and implicit (minimize the likelihood of a fall resulting from a slip or trip) goals. Efforts to infer objectives from observed behavior inevitably require computational models. These models are used to simulate the behavior that is optimal for a given objective function, and both the structure of the objective function and the corresponding costs are varied until one finds a function that produces a reasonable estimate of observed behavior [92–94]. However, these methods often consider only a limited set of potential costs and assume that humans can find optimal actions for a given cost function. We need to understand when these theories fail to predict behavior accurately and identify alternative theories that better capture the human side of human-device interactions. For reviews on habitual, feasible, good-enough, sub-optimal, and optimal motor learning and performance, see [59, 60, 95].

The promise of wearable robotic devices requires that these devices be untethered from bulky power supplies and autonomously adaptable to the varying demands faced in the real world. One of the major recent innovations in the control of wearable exoskeletons is the development of online control optimization strategies that work when walking in the real world [96]. While these strategies have yielded promising reductions in metabolic cost in young adults, it remains to be seen if these strategies work in populations with neuromotor impairments, such as people post-stroke. In addition, there is a need for continued innovation in device design to improve the likelihood that potential end-users will use these devices regularly. Computational methods can be used as part of a model-based design optimization to reduce weight and cost and potentially increase the accessibility of these innovations to diverse communities.

Similarly, ‘where’ computation happens is critical to the deployment and use of ‘smart’ assistive and rehabilitation devices. One option is the traditional von Neumann architecture, requiring a central processor and memory that takes inputs and produces outputs. Nature, in contrast, has evolved hierarchical distributed sensorimotor neural architectures, where computation happens throughout (centrally, in middleware, and ‘the edge’). This form of biological edge computing happens at subcortical, spinal, and even anatomical levels [97–99]. Therefore, successful smart neuro-assistive or neuro-rehabilitation devices (which are, in fact, a hybrid human+robot system engaged in a game-theoretic dance) would, like robots in general, do well to learn from such forms of biological edge computing for physical action.

Improving the quality of life for people with disabilities also requires that we develop more effective means by which people can navigate the digital world. The digital revolution has given us a range of powerful and relatively inexpensive devices we use to communicate with people worldwide. Although many of these devices have only existed for a short time, innovations driven by experts in human-computer interaction have resulted in intuitive user interfaces to control these devices. However, many neuromotor disorders, such as stroke, spinal cord injury, and amputation, reduce the number of options with which people can interact with the physical world. Rehabilitation engineers need to devote effort to improving accessibility to people with different levels of ability, and computational models provide a valuable means by which interfaces can be designed for efficient and inclusive use.

## Modeling ‘in-the-wild’

### Brief background

Extending impact outside of the clinic or laboratory (i.e., truly improving activities of daily living) is an essential element for effective, reliable, and personalized neurorehabilitation. Behavior ‘in the wild’ was the crucible in which evolution occurred. Thus it is ironic that, from the perspective of scientific inquiry and clinical applications, it is work and research ‘in the wild’ which has taken the longest to develop. It is only now that, for example, at-home clinical and rehabilitation applications are becoming affordable, possible, and even reimbursable in the United States via remote therapeutic monitoring mechanisms.

In contrast, clinics and laboratories have been the default controlled environments where patients can be asked to conduct standardized assessments to evaluate function and recovery. In these environments, we aim to use highly repeatable and informative assessments that can provide actionable clinical insight to guide and inform neurorehabilitation. However, once we move outside of these environments into—*‘*the wild’—we lose many of the pillars, processes, and attitudes that support traditional clinical and scientific inquiry. Executing specific motions or having highly-trained clinical hands guide an action is no longer possible nor desirable, and we must treat, control, monitor, learn, and infer from noisy data collected during variable real-work actions in non-idealized environments. Yet, being able to assess and extend neurorehabilitation into these environments is essential to support long-term function and quality of life—and the true need of our clients.

Beyond the scientific utility of in-the-wild research, we also have the social imperative of expanding services into-the-wild to increase access, including delivery of essential services to under-served populations. Transportation to/from clinic visits remains one of the largest barriers to rehabilitation care [100–103]. During the pandemic, the growth and impact of telerehabilitation demonstrated the feasibility and potential of remote assessment and monitoring to improve health outcomes [104–107].

How we got to the current model of limited in-patient and out-patient options for rehabilitation at a clinic is a function of historical, social, economic, and political influences. But we are seeing a paradigm shift, made possible by innovations in internet connectivity, wearable and ubiquitous sensors, cloud storage/analytics, and, most importantly, social and economic changes in the reimbursement landscape due to the COVID-19 pandemic that make ‘telemedicine,’ and by extension, remote therapeutic monitoring, possible and even desirable [108, 109]. In the traditional practice of out-patient neurorehabilitation, the client must travel to the clinic where they may receive one to four hours of physical or occupational therapy each week for assessment and focused training. Outside of these few hours, they may need further supports for at-home exercise programs, translating new motion patterns to activities of daily living, identifying unsafe (e.g., fall risk) scenarios, monitoring function, and guiding future in-clinic therapy sessions. There are immense opportunities for computational modeling to support and optimize neurorehabilitation in each of these scenarios—both in the clinic and at home.

New technology, like wearable sensors and environmental monitoring (e.g., via video, voice, typing, or space) can now be used to monitor and assess function in-the-wild, yet there are a dearth of tools available to leverage this data for clinical assessment or recommendations [110–113]. In the last decades, advances in wearable and ubiquitous sensing have led to extensive growth in the availability and amount of data available from daily life to potentially inform and enhance rehabilitation. This was not necessarily by design or intent. The market for devices designed exclusively for rehabilitation has often been viewed as ‘too small’ to attract serious industrial or financial investment. As a result, many neurorehabilitation systems for clinic and home use struggle to become affordable, widely used products, such as several types of rehabilitative electrical stimulators (e.g., Freehand System, BIONs, Second Sight) and exoskeletons and rehabilitation robots (ZeroG, Lokomat, Manus, Kinarm). Rather, the larger markets for military and industrial systems, and consumer products led to large-scale design and production that provided affordable, miniaturized, and widely available technologies that rehabilitation communities then adapted and adopted. A few examples (from many) are the computer mouse; graphical user interfaces; the 3D gaming wands; VR gaming goggles; global positioning system locations (GPS, Department of Defense); accelerometers (e.g,. for airbags in the automotive industry); and ultra-fast graphical processing units. This entirely non-medical and non-research ecosystem now provides sensors, communication infrastructure, algorithms, hardware, and software that can be, and has been, brought to bear on enhancing neurorehabilitation in-the-wild.

As a result, the large majority of adults in the United States and the industrialized world can afford a smartphone+smartwatch combination with accelerometers, inclinometers, GPS, cameras, heart-rate monitors, blood-oxygen saturation sensors, fast processors, and sufficient memory with sufficiently high bandwidth to monitor metrics of health and performance (e.g., step count and walking speed) or provide app-based guidance on rehabilitation exercises [114]. Recent studies have demonstrated that video-based techniques and wearables can be used to perform standardized clinical assessments [115]. For example, in Parkinson’s Disease finger tapping tasks or passive monitoring of movement characteristics can be used to tune medication dose/timing and monitor disease progression [116, 117]. Advances in robotic and haptic technology that can provide assistance or resistance, as well as virtual and augmented reality provide additional tools to develop and deploy novel neurorehabilitation approaches in the home and community [118, 119]. Computational modeling is an essential component to enable and leverage these new tools and data to guide neurorehabilitation. Machine learning often underlies these modeling methods, whether using large training datasets to prospectively monitor new patients or using unsupervised learning to identify deviations from typical or desired patterns of activity [120–122]. Physics-based modeling, such as musculoskeletal modeling and simulation, can complement and improve the accuracy of movement or exercises captured with wearable sensors [123–125]. For example, using musculoskeletal modeling and dynamic simulation can improve the accuracy of video-based techniques that estimate joint positions and movement patterns. At the population level, these techniques can evaluate the impact of novel rehabilitation techniques, differences in recovery responses, and expected trajectories of recovery or disease progression. On an individual level, personalized models can use data collected in-the-wild to customize exercise programs (e.g., adjusting challenge level), monitor daily activities (e.g., fall risk or medication responses), and provide quantitative feedback to the clinical team. The intersection of computational modeling with neurorehabilitation in-the-wild represents an exciting and high-potential area for understanding and improving outcomes, managing disease progression, and accelerating recovery.

### Commentary

The potential applications and impact of computational modeling in-the-wild to support neurorehabilitation make this an area of high priority to expand access and improve outcomes. Given the importance of expanding access and reducing burdens of rehabilitation, we were surprised and disappointed that only 15% of submissions focused on applications in-the-wild. Further, most studies focused on the sensing technologies to monitor movement and not the methods to translate results into actionable clinical insight nor tools to implement rehabilitation outside the clinic. While there is often great enthusiasm about being able to monitor and measure outside the clinic, figuring out how to bridge the gap between technology (sensors, robotics, augmented reality) and integration with rehabilitation practices is a persistent challenge. This gap contributes to continuing inequities in care.

If appropriately deployed, computational modeling can be a bridge between data, insight, and access. McGinnis’s examples of deploying multimodal sensing to collect the large datasets necessary to guide population-based and individualized insights for rehabilitation provide a compelling model for other clinics to follow and partner (see Appendix). Ideally, systems that could capture, share, and integrate data with electronic medical records across institutions would be available to create large datasets to guide future practice. Similarly, McGinnis and Scheidt provided examples of how unobtrusive sensing can extend measurements outside the clinic to monitor falls, disease progression, and mental health. Song and Collier demonstrated how complementing these sensing techniques with cloud computing, theoretical models, and neuromechanical simulations can deepen our understanding of the mechanisms driving rehabilitation responses. Integration of complex systems will be required to leverage these advances towards personalized and optimized neurorehabilitation. Ultimately, we would envision a system that could quantify the specific mechanisms contributing to an individual’s functional capacity, use that insight to develop a personalized neurorehabilitation plan that minimizes patient, caregiver, and clinician burden, repeatedly monitor relevant digital biomarkers outside of the clinic, integrate information into clinically meaningful and interpretable charts in a patient’s electronic medical record, and use this monitoring to continuously adapt and optimize rehabilitation and predict long-term outcomes.

Moving towards this level of personalization and precision will require development of new methods to induce neuroplasticity, personalize care, and improve human-device interaction to support rehabilitation goals—the other focus areas of this conference. We recognize that these advancements also are in opposition to realities of reimbursement and provision in the American healthcare system. At the most basic level, we need to determine the methods to reimburse and capture the new sources of data that will be essential to drive rehabilitation. While we can schedule and reimburse for a session of physical therapy or an MRI, how and when to reimburse for the use of a wearable sensor or robotic device deployed in the home, and how to provide the technical and analytical expertise to integrate the data from these sensors into the clinical routine remain open challenges. Beyond implementation and reimbursement, the more complex challenges will require figuring out how to ensure patient privacy, develop equitable and inclusive algorithms, and responsibly monitor individuals in-the-wild. New standards need to be developed alongside technology to enable safe and effective neurorehabilitation that leverages computational modeling for deployment in-the-wild.

## Conclusions

### Key insights

The conference brought concrete, cutting-edge examples of how computational modeling can provide foundational insights about data that cannot be obtained experimentally, and support the formulation of useful hypotheses and the design of assistive technology and other innovative technology that can accelerate and optimize neurorehabilitation.

Computational models are, after all, hypotheses formulated as mathematical constructs—be they statistical black or gray boxes, or mechanistic paradigms. As per the scientific method, observations and experiments (two forms of data) are crucial for proper hypothesis development and testing. Furthermore, in the clinical realm, computational models are a means to transform information into actionable insights and therapeutic decisions. There were four common threads across the Focus Areas that underpin future needs to create useful data and models for neurorehabilitation:

(i) The need to capture and curate appropriate and useful data necessary to develop, validate, and deploy useful computational models. This step is critical to models for applications that span from classification of clients (as with the ENIGMA Stroke Recovery Working Group to use structural neuroimaging to classify clients), to real-time online use of data (as in human-in-the-loop systems).
(ii) The need to create multi-scale models that span the personalization spectrum from individuals to populations, and from cellular to behavioral levels. Figure 2 emphasizes this point as most of the work presented can be placed on this two-dimensional spectrum to clearly identify the types of questions a model is addressing, and the generalizability of its findings. This was particularly critical for models related to adaptation and plasticity, which can occur across all ranges of scale and number.
(iii) A strong case was made to pursue means to extract as much information from available data (meta-learning), while requiring as little data as possible from each client. This is because there are important ethical, practical, and algorithmic limitations on just how much, and what kind of, data a given individual can contribute to the development or tuning of a model or a human-in-the-loop system.
(iv) The insistence on leveraging readily available sensors and data systems to push model-driven treatments away from the lab, and into the clinic, home, and workplace. However, translating knowledge into action is a longstanding and difficult challenge in medical research [126, 127]. The era of the Internet-of-Things and the Internet-of-Medical-Things, nevertheless, is here to stay—even if it faces skepticism and resistance [128]. We should embrace it. In addition to the traditional need to translate research into action in the form of devices and methods, we now also have the robust public debate about the costs and dangers of bringing a relative newcomer, Artificial Intelligence (AI) into clinical practice. The conference, however, brought into clear focus the costs of not using data-driven computational modeling for healthcare. Simply put, there is ample opportunity to personalize care by using existent technologies ‘in-the-wild’ based on consumer products that can have real positive and ethical impact to neurorehabilitation at low cost, deployable at scale, and without compromising privacy. But these efforts need to be done in close collaboration with clinicians and clients, while avoiding the temptation to decouple data from physiology.

### The way forward

In addition to the Commentary made for each section above, it is important to reiterate that very recent developments in transformers and their variants (when feasible) will provide exciting novel opportunities in all Focus Areas (Table 1) and across scales (Fig. 2) that we can scarcely imagine at this point. Notwithstanding that transformers are hugely ‘data-hungry’ and require large training times, they can find applications in neurorehabilitation. In ‘Modeling for Personalization’ applications, we mentioned the example where they can help us bridge the gap between generic and patient-specific computational models for, say, tumor detection in a given patient based on thousands of structural MR images [64]. Transformers and their foundation on the ‘attention mechanism’ are also finding traction in other relevant applications where large datasets are available. For example, ‘Modeling human-device interactions’ provides a fertile ground where transformers can learn from large sets of EEG or ElectroCorticoGraphy (ECoG) signals [129, 130] collected over days of recordings in patients interacting with neuroprosthetics or neuromodulation systems like deep brain stimulation (DBS). Similarly, wherever wearables or markerless video provide large sets of movement data. Thus ‘Modeling ‘in-the-wild” will be able to provide generic and personalized movement patterns and syntax that can be readily used to track adaptation and plasticity in human environments [131, 132].

It is our firm belief that the open, sincere, and robust discussion at the conference, and the resulting videos, journal articles, and ideas sparked from collaborative conversations serve as strong medicine against these maladies. The conference enabled and forced us to examine the nature and appropriate uses of existing computational techniques to now refine them or develop new ones. And they confronted us with the need for a tight closed-loop interaction with the functional and clinical reality of our health care professionals and clients to focus our computational neurorehabilitation work on useful, urgent, and relevant problems and solutions. We look forward to the next few years to reconvene the next iteration of this conference to re-assess the progress in these critical Focus Areas and bring to fruition the many opportunities to catalyze progress in neurorehabilitation. We stand on the shoulders of giants, and the best is yet to come.

## Data Availability

Permalink: (https://web.archive.org/save/http://dare2023.usc.edu).

## Appendix: overview of key insights made in presentations by the speakers

### Modeling adaptation and plasticity

*Sook-Lei Liew: using large heterogenous neuroimaging datasets to model stroke rehabilitation outcomes* Recovery after stroke is highly variable due to heterogeneity in direct stroke damage (e.g., lesion size, location), and resulting secondary neural damage, as well as due to differences in how these neurologic changes translate into behavioral consequences. Large, diverse datasets are thus important to provide sufficient data to allow for greater generalizability of computational models in stroke. Addressing this need, the ENIGMA Stroke Recovery Working Group harmonizes neuroimaging and behavioral data from research groups worldwide to generate large, heterogeneous datasets of structural neuroimaging for computational modeling approaches, resulting in well-powered and generalizable findings about the relationship between post-stroke sensorimotor outcomes and brain characteristics, such as lesion size, location, brain age, atrophy, and connectivity.

*Amy Orsborn: co-adaptive therapies to shape user learning via plasticity-aware decoding* Technologies that interface with the nervous system produce plasticity. For example, how neural activity relates to limb movement—the brain’s encoding of it—can change as someone practices with a brain-computer interface (BCI). We find that changes in the brain’s encoder are strongly influenced by the decoding algorithm used in a BCI. These interactions create complex bi-directional dynamics (e.g., closed-loop, dynamical coupling, and game-theoretic interactions such as predator-prey). Thus uni-directional computational methods that ignore plasticity may improve short-term BCI performance at the potential cost of long-term performance. Yet, these bi-directional interactions also open new opportunities to shape the nervous system for rehabilitation applications. Achieving this goal will require new computational approaches capable of capturing dynamic and non-stationary interactions between the nervous system and a device, such as those in the field of game theory. [133]

*Adam Roth: the role of reinforcement-based and error-based processes on exploratory motor behavior in neurologically intact and Parkinson’s disease* Exploration is critical when attempting to re-learn functional motor skills following a neurological disorder. Yet we know little on how error-based and reinforcement-based processes interact to influence motor behavior. Here, Roth and colleagues designed three experiments and a computational model to investigate the unique and interacting roles of reinforcement feedback and error feedback on motor exploration. Reinforcement-based and error-based feedback respectively boost and suppress exploration, while when together they oppose one another to result in moderate exploratory motor behavior. In contrast, participants with Parkinson’s disease, who have known deficits to reinforcement-based neural circuits—on account of disruption of dopamine-dependent neural circuitry—showed less exploration with reinforcement feedback compared to neurologically intact age-matched controls; but similar levels of exploration when given error feedback or both reinforcement and error feedback simultaneously. Such neural circuitry involved in error-based feedback could, therefore, be exploited for neurorehabilitation to improve outcomes in Parkinson’s disease.

*Dulce Mariscal: characterization of locomotor adaptation and generalization dynamics from high-dimensional neuromuscular data* Humans can adapt their gait to compensate for changes in environmental demands, and generalize learned movements from one situation to another. One way to study locomotor adaptation is by exposing participants to split-belt treadmill walking and contrasting the adaptation effects (i.e., after-effects) that participants exhibit in the same (treadmill) or different (overground) contexts from the adaptation. We used a data-driven approach to determine the processes that underlie the adaptation of muscle activity. Our results suggest that reactive and contextual patterns contribute to the evolution of neuromuscular patterns during split-belt walking. However, the generalization of these patterns to walking without the training device is much smaller, contributing to the smaller kinematic after-effects previously reported while walking overground. These analyses provide insights into locomotor adaptation features beyond those drawn from traditional kinetic or kinematic analyses [134–136].

*Felix Schwock: a novel graph diffusion framework for estimating neural communication towards personalized neurorehabilitation* Most neurological diseases are associated with altered patterns of neural communication; however, their specific changes due to disease or subsequent rehabilitative treatments could be better understood. As a step towards that, we propose a new computational framework for estimating dynamic network level neural communication by modeling the evolution of neural activity as a parameterized graph diffusion process [137]. To demonstrate the utility of our framework for neurorehabilitative applications, we have applied it to electrocorticography recordings from the sensorimotor cortex of a macaque monkey that underwent focal ischemic lesioning and acute electrical stimulation in the ipsilesional hemisphere [138]. We found that stimulation in the acute phase after lesioning caused an increase in neural communication near the stimulation location in both hemispheres that was not observed when analyzing classical measures of neural communication such as coherence or Granger causality. This framework opens opportunities for studying network level neural communication with different experimental setups on various spatiotemporal scales, thus, supporting the development of personalized and adaptive treatments for various neurological disorders by tailoring treatment protocols to an individual’s network dynamics.

*Ariel Edward Hight: neuroplasticity and improved speech perception in cochlear implant users* Cochlear implants are auditory prostheses that restore hearing and speech perception to humans with severe to profound hearing loss. These devices work by inserting as many as 22 electrodes into the cochlea, where sound is normally transduced into neural activity. Patterned electrical stimulation of the auditory nerve by implanted electrodes induces significant remapping of sensory codes for hearing [139], and attaining significant speech perception (without lip-reading) can occur over weeks, months, and in some cases years [140–142]. Our results indicate that discriminating spectro-temporal cues in human cochlear implant users improves over initial implant use, paralleling periods of rapid improvement in speech perception. Furthermore, we have developed a system for behavioral and physiological studies of cochlear implants in deafened rats [143], and are studying mechanisms of neuromodulation and plasticity for hearing restoration, including new experiments asking if and how vagus nerve stimulation might help improve perceptual learning with cochlear implants[144].

### Modeling for personalization

*Steve Collins: personalizing exoskeleton assistance in the real world: learning models of human–device interaction to leave the lab behind* Human-in-the-loop-optimization is a technique for personalizing assistive device characteristics based on rapidly generated, local models of user response. The approach has been successful at augmenting mobility for healthy adults using exoskeletons, leading to the largest improvements in speed and energy economy to date under real-world conditions [96]. It is critical to extend these techniques to new outcomes, such as balance and pain, and populations with mobility loss, including older adults and individuals with chronic stroke.

*Katherine Saul: integrating medical imaging and iterative modeling approaches for personalized simulation* Although we have unprecedented computing power to personalize musculoskeletal models, data to inform models are limited. It is difficult or impossible to directly and noninvasively measure many of the numerous musculoskeletal parameters; instead we are currently limited to measurements in cadaveric specimens, small populations, or small numbers of muscles that do not span the diversity of the population or physiology. Judicious personalization approaches require one to think strategically about the desired scientific outcome: is it necessary to understand the underlying mechanics and parameters, or is it necessary to predict the desired behavior most accurately? Detailed personalization schemes that seek to personalize all parameters may obscure fundamental biomechanics in the same way clinical measures can, and thus sensitivity analyses may be more appropriate [145–147]. For intuitive control approaches for human-machine interfaces, personalized physics-based models that tune parameters to best control intended function may be most appropriate [148, 149], but these may not directly elucidate the underlying parameters or biomechanics. When choosing a personalization approach, researchers are encouraged to engage clinicians early in the design of the research question, choose a personalization scheme carefully to capture major clinical features, and choose simulation outcomes that are parallel to clinical assessments to enhance interpretation, translation, and validation.

*James Patton: optimal personalized designs of spring-network devices* Exoskeletal devices have demonstrated their potential for assistance, therapy, and even prosthetic applications. Importantly, much can be accomplished with passive structures using a network of impedance elements that can approximate nearly any desired fields. For example, a leg exoskeleton with the right number and placement of layered linear spring elements can approximate the required torques otherwise provided by muscle, and structural optimization methods can personalize the design of exoskeletons for neurorehabilitation. In fact, it is possible to optimize the design of a wearable device, the ExoNET, which uses elastic elements to provide assistive torques to individuals with motor deficits during gait. Potential benefits of this approach are reducing muscle engagement and metabolic cost of walking while using optimization algorithms and sensitivity analysis to improve the device’s performance. We also explore how computational modeling can facilitate the custom-design optimization for individuals post neurological impairments. Such optimization algorithms can be used to design this network of multijoint tension elements for gait and balance, in individuals with neurological or musculoskeletal conditions. Sensitivity analysis with co-varying parameters can identify the parameter subspace that is most influential on the torque. These methods map high-dimensional spaces to lower dimensional manifolds of the parameter space, resulting in a subspace manifold to allow rapid human-in-the-loop optimization (HIL) for a personalized, safe, and easy-to-use device that can assist with movement and improve gait and balance.

*Guillaume Lajoie: rapidly personalizing models of stimulation-evoked neural responses with meta-learning* Due to variability arising from placement of stimulation devices, underlying neuroanatomy and physiological responses to stimulation, it is essential that neurostimulation protocols are personalized to maximize efficacy and safety. Building such personalized protocols would benefit from accumulated information in increasingly large datasets of other individuals’ responses. To address that need, a meta-learning family of algorithms can be used to conduct few-shot optimization of key fitting parameters of physiological responses in new individuals [150]. This meta-learning framework is general and can be adapted to any input-response neurostimulation mapping problem.

*Jessica Allen: age- and stroke-related impairments in the neuromuscular control of dynamic balance during walking* Falls due to a loss of balance during walking are a primary cause of injury in older adults and individuals with neurological deficits [151, 152]. Recent work implicates an innovative mechanism of neuromuscular control that may be critical for successful walking function [153–155]. Specifically, embedding the control of balance into the muscle coordination for walking is associated with better walking function. Moreover, this relationship was found in both neurologically impaired and intact individuals, suggesting that it may represent a general neuromuscular strategy contributing to the maintenance of balance while walking and may serve as an effective target for individualized neurorehabilitation design and prescription.

*David Lin: modeling brain-behavior relationships after stroke to advance neurorehabilitation* A series of clinical research projects derived from a natural history study of upper extremity motor recovery after stroke, features the prospective collection of data at the point of care (in-hospital, in outpatient clinics) across the stroke continuum of care (Stroke Motor Rehabilitation and recovery Study, NCT03485040). Findings from that data collection effort have revealed that (1) the corticospinal tract derived from clinical imaging can be used to predict upper extremity motor recovery after stroke [156]; (2) cognitive demands influence upper extremity motor performance after stroke [157]; (3) broad, disability focused outcomes (i.e. the modified Rankin scale) do not necessarily capture changes in impairment or function in the first 90 days after stroke [158]; (4) patient-reported and performance-based outcomes have distinct neuroanatomic correlates (manuscript in preparation); and (5) there are distinct neural circuits for proximal versus distal upper extremity motor control [159]. Importantly, these projects have all featured critical interdisciplinary collaborations between neurologists, neuroscientists, therapists, and engineers. Overall, those findings highlight the value of outcome measure collection at the clinical point-of-care, providing fundamental insights that can guide research trials and clinical rehabilitation. Outcome measures in neurorehabilitation have distinct meanings and neurologic underpinnings that need to be thought about carefully in the context of computational modeling.

### Modeling human–device interactions

*Jeremy D. Brown: understanding the utility of haptic feedback in teleoperated and assistive robots* For assistive robotic devices such as prosthetic limbs, the absence of haptic information leads to a decrease in task performance and an increase in cognitive load [160]. This knowledge provides useful insight into the possible consequences of impaired haptic sensation in the biological limb. Yet, our current understanding of sensory impairment and sensory recovery is limited. Robotic technologies are well-poised to help fill this knowledge gap by providing robust and reliable assessments of sensory function [161].

*Michelle Johnson: toward automated assessment of human–human and human–robot interaction for neurorehabilitation* There is an increased need for rehabilitation to occur outside traditional settings and in real-world environments. Fundamental to successful neurorehabilitation are effective human–human interactions between the patient and therapists in clinical and non-clinical settings. Often these interactions are done within billable rehabilitation tasks and are embedded with a clinical need for in-person or remote automated assessment of persons (adult, child or infant) with motor and/or cognitive disabilities. There are several efforts to develop algorithms to objectively measure motor and cognitive behaviors while people perform tasks with an affordable haptic therapy robot, a mobile social robot with telepresence, and a toy robot. This allows the development of computational models to study both human–human interactions and human–robot interactions to automatically assess motor delay in infants, automatically classify interaction behaviors and enable more personalized use of robots in rehabilitation. Importantly, there are key opportunities and needs to advance these computational models.

*Kayla Pariser: computational treatment design of adaptive treadmill controllers* Computational design of treatments using predictive simulations may allow for more efficient selection of optimal rehabilitation compared to fatiguing trial-and-error experiments. Thus it is important to develop and evaluate a predictive simulation framework to estimate changes in gait with various novel adaptive treadmill controllers and to determine who will benefit from which controller. The adaptive treadmill simulation framework successfully predicted similar changes in walking speed, propulsive mechanics, kinematics, and spatiotemporal parameters that we observed experimentally with the different treadmill controllers. This lays the foundation for how computational modeling can inform design of rehabilitation protocols and estimate cause-and-effect for how individuals will respond to novel therapeutic interventions.

*Zachary Lerner: modeling human-exoskeleton interactions to predict neuromuscular engagement during walking with targeted resistance* Step-by-step assessment of muscle recruitment during walking with resistive exoskeletons would allow for the development of automated biofeedback systems aimed at incentivizing user engagement during gait training [162]. Supervised machine learning techniques may be used to predict neuromuscular engagement of the ankle plantar flexors muscles in real-time during walking with ankle exoskeleton resistance in individuals with cerebral palsy . A recent pilot study deploying the real-time predictions during gait training demonstrate that data-driven artificial neural networks can be used to improve the efficacy of robot-aided rehabilitation [163].

*Brandon Peterson: a design-optimization framework for compliant implanted prostheses that restore joint function* When human joints are damaged due to injury or disease, mobility can become severely limited and painful [164]. While most major joints can be reconstructed, conventional implants do not last forever—typically due to failure mechanisms involving aseptic loosening and mechanical instability. Thus, there is need for, and opportunity in, a fundamental shift in the design framework for joint-replacing implants, centered around compliant mechanisms. These mechanisms guide motion through the elastic deformation of flexible elements, and can be designed to be both frictionless and inherently stable [165].

*Natalija Katic Secerovic: modeling afferent tactile responses from the sole of the foot* Cutaneous feedback from the foot sole is crucial for gait and balance control [166]. Electrophysiological recordings provide insights into how afferent populations encode tactile information [167]. However acquiring such recordings is challenging and restricted to stationary conditions. A promising approach to this challenge is the FootSim model that simulates neural spiking responses to arbitrary mechanical stimuli from the combined population of all four types of mechanoreceptors innervating the foot sole [168]. From neuroscientific perspective, it can be exploited for unveiling the afferent activation in dynamic situations, overcoming the limitations of currently available recording techniques, while from the neurotechnological point of view the model can be used for neuroprosthetic applications as in-silico tool for designing biomimetic stimulation paradigms.

### Modeling ‘in-the-wild’

*James Cotton: portable, in-clinic, video-based analysis of gait impairments* Numerous methods have recently emerged for monitoring gait in the clinic and community, including fixed and wearable sensors, markerless motion capture and video-based analysis. Processing, analyzing, interpreting and using these data presents numerous challenges, including limited generalization of pretrained models like keypoint detectors and activity recognition algorithms to people with disabilities, whose limbs and movements may look different than able-bodied individuals in the training data [169]. In-the-wild recordings also raise important ethical issues of AI fairness for people with disabilities and highlight the technical need to robustly validate these systems on clinical populations. After addressing these challenges, we show that it should be possible to routinely and quantitatively analyze the gait of many people seen in rehabilitation clinics and performing in-home therapy. The resulting large datasets also present a new set of challenges for identifying phenotypes and subgroups who would benefit from different treatment plans under a precision-rehabilitation framework, and the opportunities to incorporate causal inference in our computational models to bridge across levels within the International Classification of Function (ICF) framework, and across time between providing rehabilitation interventions and long-term functional outcomes and quality of life.

*Ryan McGinnis: modeling to enable personalized and preventative digital medicine in-the-wild* The advent of conformal, skin worn sensors has enabled unobtrusive continuous monitoring of patients outside of traditional laboratory or clinical environments. These emerging sensors with advanced data analysis pipelines form a digital biomarker discovery platform [170, 171]. Such platforms detect activities of daily living, quantify how patients engage in those activities, and identify potential biomarkers of symptoms or disease that can then be monitored over time to inform assessment and efficacy of interventions. We have shown this platform can be used for studying biomarkers of fall risk in persons with multiple sclerosis that can be extracted from everyday walking, postural transitions, and standing [170, 171]. This allows identifying several key areas of consideration for advancing remote patient monitoring, including selecting appropriate approaches for data aggregation (e.g., averages alone are probably not sufficient), considering appropriate monitoring periods (e.g., period depends on population and parameter), and the need for careful validation to ensure these technologies are fit for purpose [172].

*Robert Scheidt and Kim Bassindale: Souvenir: a case study of challenges and opportunities in the integration of computational intelligence with wearable rehabilitation technology in acute care and at-home settings* Exercise is medicine in physical rehabilitation [173]. Motivating patients to actually do prescribed exercises on their own is a practical challenge facing all practicing clinicians. We propose that low-cost wearable mobile health (mHealth) systems have potential to encourage increased use of the more-affected arm throughout all phases of recovery after stroke—without significantly increasing caregiver burden. To test this idea, we developed a system (called *Souvenir*) to provide salient cues periodically reminding stroke survivors to perform their prescribed exercises. The system comprises two off-the-shelf wrist-worn motion trackers, a smart phone, an easy-to-use custom app, and a set of three ‘progressive challenge’ exercises developed by practicing clinicians. The system also offers the possibility for increased clinician access to motion data throughout the day to assess exercise adherence and performance. Preliminary testing shows that the progressive exercises had the intended effect as stroke survivors used their more-affected arm most when cued to move it independently, and least when instructed to simply tap it with their less affected arm. Although that study was not designed to test efficacy, this pattern of arm use appeared to generalize to non-cued, silent monitoring periods, suggesting that the effect of the cued exercises on the more-affected arm use may bleed over into increased spontaneous performance of everyday activities. As currently implemented, Souvenir’s reliance on manual progression through the exercise challenge levels may not yield optimal transfer of increasing hemiparetic arm use from cued exercise intervals to periods without cues. This limitation poses an opportunity for algorithmic intelligence to model the user’s state and adjust the exercise challenge on the fly to maximize beneficial transfer. How best to model user state and motivate behavioral change remains an open question because exercise compliance is dependent on complex and interrelated psychological factors including depression, motivation, fatigue, and perceptions of self-efficacy.

*George Collier: using big data and a variety of modeling approaches to advance rehabilitation care* We leveraged a modern multiparadigm, big data analytics platform built on Azure component systems to analyze very large data sets. A diverse set of data sources was integrated into the platform. This allowed us to build highly successful mathematical, machine learning and theoritical models of human behavior and physiology while performing rehabilitation focused exercises.

*Seungmoon Song: modeling in-the-wild effects of gait assistive devices through neuromechanical simulations and deep reinforcement learning* Over the past years, we have developed neuromechanical simulations that capture various aspects of human locomotion [174, 175]. Our ultimate aim is to create digital motor clones that can supplement human subject experiments in the development and testing of assistive devices and rehabilitation treatments. To achieve this goal, our ongoing research focuses on two main areas: personalizing musculoskeletal and motor control models to individual human subjects, and utilizing deep reinforcement learning techniques to devise methods for developing physiologically plausible controllers capable of managing a wide array of movements in rehabilitation-related settings [176].

*Haylie Miller: measurement of neurodivergent visuomotor skills in-the-wild* Measurement of human visuomotor behavior in-the-wild requires integration and interpretation of multimodal data during naturalistic tasks. Our team uses a novel visuomotor assessment protocol and our VMIntegration algorithm (NSF SMA-1514495; NIH K01-MH107774) to precisely measure postural control problems and their relation to sensory processing in neurodivergence (e.g., autism, ADHD, dyspraxia) [177, 178]. Our visuomotor assessment paradigm yields multimodal data that is fed back into both hypothesis-driven and data-driven computational models of visuomotor integration and postural control to identify clinically-significant problems, phenotypes within and between diagnostic conditions, and high-yield intervention targets. Our approach can be used in community settings with neurotypical and neurodivergent children, adolescents, and adults across a wide range of abilities [179], reducing barriers to identification of visuomotor problems that can negatively impact daily living skills and quality of life [180, 181].

## Footnotes

1 RESNA was started in August 1979 at a meeting of the Inter-Agency Conference on Rehabilitation Engineering when participants Douglas Hobson, Colin McLaurin, James Reswick, Anthony Staros, and Joseph Traub offered a resolution to form the ‘Rehabilitation Engineering Society of North America’ first housed at Rancho Los Amigos Medical Center in Los Angeles, CA.
